# (1R*,2S*,4S*,5R*)-Cyclo­hexane-1,2:4,5-tetra­carb­oxy­lic dianhydride

**DOI:** 10.1107/S1600536812003571

**Published:** 2012-02-04

**Authors:** Akira Uchida, Masatoshi Hasegawa, Eiichiro Takezawa, Shinya Yamaguchi, Atsushi Ishikawa, Takashi Kagayama

**Affiliations:** a Department of Biomolecular Science, Faculty of Science, Toho University, 2-2-1 Miyama, Funabashi, Chiba 274-8510, Japan; b Department of Chemistry, Faculty of Science, Toho University, 2-2-1 Miyama, Funabashi, Chiba 274-8510, Japan; c Department of Research and Development, Gas Chemical Division, Iwatani Industrial Gases Corporation Ltd, 10 Otakasu-cho, Amagasaki, Hyogo 660-0842, Japan

## Abstract

The title compound, C_10_H_8_O_6_, a promising raw material to obtain colorless polyimides which are applied to microelectronic and optoelectronic devices, adopts a folded conformation in which the dihedral angle between the two anhydro rings is 55.15 (8)°. The central six-membered ring assumes a conformation inter­mediate between boat and twist-boat. In the crystal, mol­ecules are linked by weak C—H⋯O inter­actions, forming a layer parallel to the *bc* plane.

## Related literature

For microelectronic applications of the present compound, see: Ando *et al.* (2010 ▶). For background to polyimides, see: Hasegawa *et al.* (2007 ▶, 2008 ▶); Hasegawa & Horie (2001 ▶). For a related structure, see: Uchida *et al.* (2003 ▶).
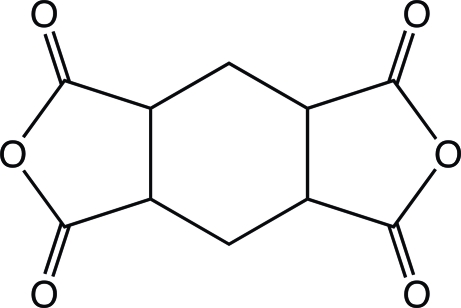


## Experimental

### 

#### Crystal data

C_10_H_8_O_6_
*M* *_r_* = 224.16
Monoclinic, 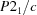

*a* = 12.167 (2) Å
*b* = 7.1380 (14) Å
*c* = 10.626 (2) Å
β = 90.12 (3)°
*V* = 922.8 (3) Å^3^
*Z* = 4
Mo *K*α radiation
μ = 0.14 mm^−1^
*T* = 296 K
0.51 × 0.42 × 0.42 mm

#### Data collection

Bruker APEXII CCD area-detector diffractometer
Absorption correction: multi-scan (*SADABS*; Sheldrick, 1996 ▶) *T*_min_ = 0.934, *T*_max_ = 0.945
6648 measured reflections
2285 independent reflections
2015 reflections with *I* > 2σ(*I*)
*R*_int_ = 0.026

#### Refinement

*R*[*F* ^2^ > 2σ(*F* ^2^)] = 0.045
*wR*(*F* ^2^) = 0.140
*S* = 1.00
2285 reflections
146 parameters
H-atom parameters constrained
Δρ_max_ = 0.30 e Å^−3^
Δρ_min_ = −0.18 e Å^−3^

### 

Data collection: *APEX2* (Bruker, 2007 ▶); cell refinement: *SAINT-Plus* (Bruker, 2007 ▶); data reduction: *SAINT-Plus*; program(s) used to solve structure: *SHELXS97* (Sheldrick, 2008 ▶); program(s) used to refine structure: *SHELXL97* (Sheldrick, 2008 ▶); molecular graphics: *PLATON* (Spek, 2009 ▶) and *ORTEPIII* (Burnett & Johnson, 1996 ▶); software used to prepare material for publication: *SHELXL97* and *PLATON*.

## Supplementary Material

Crystal structure: contains datablock(s) global, I. DOI: 10.1107/S1600536812003571/is5043sup1.cif

Structure factors: contains datablock(s) I. DOI: 10.1107/S1600536812003571/is5043Isup2.hkl

Supplementary material file. DOI: 10.1107/S1600536812003571/is5043Isup3.mol

Supplementary material file. DOI: 10.1107/S1600536812003571/is5043Isup4.cml

Additional supplementary materials:  crystallographic information; 3D view; checkCIF report

## Figures and Tables

**Table 1:** Hydrogen-bond geometry (Å, °)

*D*—H⋯*A*	*D*—H	H⋯*A*	*D*⋯*A*	*D*—H⋯*A*
C1—H1⋯O3^i^	0.98	2.40	3.3384 (19)	159
C3—H3*B*⋯O6^ii^	0.97	2.58	3.429 (2)	146

Symmetry codes: (i) 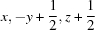; (ii) 
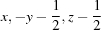
.

**Fig. 1.**
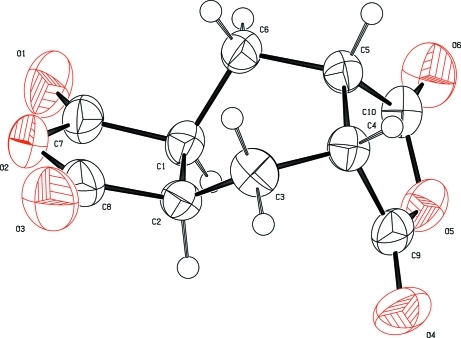
The molecular structure of the title compound, showing displacement ellipsoids at the 50% probability level.

**Fig. 2.**
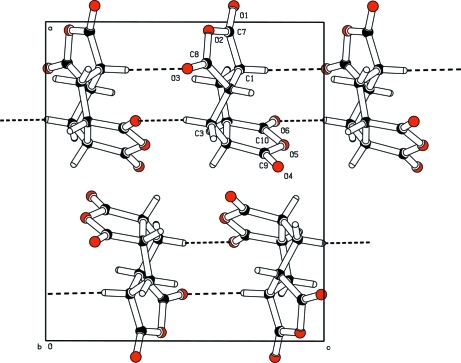
The crystal packing of the title compound viewed along the b axis. The dashed lines indicate C—H···O intermolecular interactions.

## supplementary crystallographic information

### Comment

Aromatic polyimides (PI) have been widely applied to microelectronic and optoelectronic devices for their reliable combined properties: considerably high glass transition temperatures (*T*_g_), non-flammability, and good dielectric and mechanical properties (Ando *et al.*, 2010). Conventional aromatic PI films are intensively colored on the basis of charge-transfer (CT) interactions (Hasegawa & Horie, 2001). However, the coloration often disturbs optical applications of PIs. Recently, there is a strong demand that further lightens the total weights of flat panel displays by replacing fragile inorganic glass substrates (~400 µm thick) by plastic substrates (~100 µm thick). However, it is not easy to develop the practically useful plastic substrates simultaneously possessing excellent optical transparency and sufficient heat resistance (*T*_g_'s > 250 °C) for the device fabrication processes such as inorganic transparent electrode deposition. The most effective strategy for completely erasing the significant PI film coloration is to use non-aromatic (cycloaliphatic) monomers either in tetracarboxylic dianhydride or diamine, thereby the CT interactions are inhibited. Our previous work illustrated that the equimolar polyaddition of *cis*, *cis*, *cis*-1,2,4,5-cyclohexanetetracarboxylic dianhydride (H-PMDA), synthesized by hydrogenation of pyromellitic dianhydride (PMDA), and some diamines indeed led to colorless PIs with very high *T*_g_'s (Hasegawa *et al.*, 2007). However, the obtained PI films were very brittle in some cases owing to poor chain entanglement caused by insufficient molecular weights of the resultant PIs, which come from the insufficient reactivity of H-PMDA with diamines. The low reactivity of H-PMDA can be explained in terms of its steric structure (Uchida *et al.*, 2003). In order to solve this crucial problem, we developed another H-PMDA isomer, *i.e.*, 1*R**, 2S*, 4S*, 5*R**-cyclohexanetetracarboxylic dianhydride (H"-PMDA) (Hasegawa *et al.*, 2008). The present work reports the crystal structure of this compound.

### Experimental

H"-PMDA was synthesized as follows (Hasegawa *et al.*, 2008); PMDA was hydrolyzed with a NaOH aqueous solution. The pyromellitic acid tetrasodium salt formed was hydrogenated in a high-pressure hydrogen atmosphere in the presence of a ruthenium catalyst, and neutralized with conc. HCl. The tetracarboxylic acid obtained was isomerized by dehydrating with acetic anhydride at a precisely controlled temperature. Crystals of the title compound suitable for X-ray analysis were obtained from an acetic anhydride solution.

### Refinement

All H atoms were placed in geometrical positions (C—H = 0.98 and 0.97 Å for CH and CH_2_, respectively) and constrained to ride on their parent atoms with *U*_iso_(H) = 1.2*U*_eq_(C).

### Crystal data

**Table:**

C_10_H_8_O_6_	*F*(000) = 464
*M**_r_* = 224.16	*D*_x_ = 1.613 Mg m^−^^3^
Monoclinic, *P*2_1_/*c*	Mo *K*α radiation, λ = 0.71073 Å
Hall symbol: -P 2ybc	Cell parameters from 3549 reflections
*a* = 12.167 (2) Å	θ = 2.5–28.3°
*b* = 7.1380 (14) Å	µ = 0.14 mm^−^^1^
*c* = 10.626 (2) Å	*T* = 296 K
β = 90.12 (3)°	Block, colorless
*V* = 922.8 (3) Å^3^	0.51 × 0.42 × 0.42 mm
*Z* = 4	

### Data collection

**Table:**

Bruker APEXII CCD area-detector diffractometer	2285 independent reflections
Radiation source: fine-focus sealed tube	2015 reflections with *I* > 2σ(*I*)
Graphite monochromator	*R*_int_ = 0.026
φ and ω scans	θ_max_ = 28.3°, θ_min_ = 3.3°
Absorption correction: multi-scan (*SADABS*; Sheldrick, 1996)	*h* = −16→16
*T*_min_ = 0.934, *T*_max_ = 0.945	*k* = −9→9
6648 measured reflections	*l* = −7→14

### Refinement

**Table:**

Refinement on *F*^2^	Secondary atom site location: difference Fourier map
Least-squares matrix: full	Hydrogen site location: inferred from neighbouring sites
*R*[*F*^2^ > 2σ(*F*^2^)] = 0.045	H-atom parameters constrained
*wR*(*F*^2^) = 0.140	*w* = 1/[σ^2^(*F*_o_^2^) + (0.0954*P*)^2^ + 0.121*P*] where *P* = (*F*_o_^2^ + 2*F*_c_^2^)/3
*S* = 1.00	(Δ/σ)_max_ < 0.001
2285 reflections	Δρ_max_ = 0.30 e Å^−^^3^
146 parameters	Δρ_min_ = −0.18 e Å^−^^3^
0 restraints	Extinction correction: *SHELXL97* (Sheldrick, 2008), Fc^*^=kFc[1+0.001xFc^2^λ^3^/sin(2θ)]^-1/4^
Primary atom site location: structure-invariant direct methods	Extinction coefficient: 0.057 (8)

### Special details

**Table:**

Geometry. All e.s.d.'s (except the e.s.d. in the dihedral angle between two l.s. planes) are estimated using the full covariance matrix. The cell e.s.d.'s are taken into account individually in the estimation of e.s.d.'s in distances, angles and torsion angles; correlations between e.s.d.'s in cell parameters are only used when they are defined by crystal symmetry. An approximate (isotropic) treatment of cell e.s.d.'s is used for estimating e.s.d.'s involving l.s. planes.
Refinement. Refinement of *F*^2^ against ALL reflections. The weighted *R*-factor *wR* and goodness of fit *S* are based on *F*^2^, conventional *R*-factors *R* are based on *F*, with *F* set to zero for negative *F*^2^. The threshold expression of *F*^2^ > σ(*F*^2^) is used only for calculating *R*-factors(gt) *etc*. and is not relevant to the choice of reflections for refinement. *R*-factors based on *F*^2^ are statistically about twice as large as those based on *F*, and *R*- factors based on ALL data will be even larger.

### Fractional atomic coordinates and isotropic or equivalent isotropic displacement parameters (Å^2^)

**Table:**

	*x*	*y*	*z*	*U*_iso_*/*U*_eq_	
O1	1.05049 (8)	−0.0274 (2)	0.67601 (13)	0.0712 (4)	
O2	0.97350 (9)	0.22073 (16)	0.58580 (11)	0.0583 (3)	
O3	0.85376 (13)	0.43198 (17)	0.50962 (13)	0.0773 (4)	
O4	0.54696 (9)	0.16073 (17)	0.83493 (12)	0.0642 (3)	
O5	0.61479 (8)	−0.12795 (16)	0.85526 (9)	0.0504 (3)	
O6	0.68964 (10)	−0.40914 (17)	0.82316 (12)	0.0662 (4)	
C1	0.85268 (9)	0.02100 (17)	0.69704 (11)	0.0371 (3)	
H1	0.8492	0.0003	0.7881	0.045*	
C2	0.79051 (10)	0.20009 (16)	0.66127 (11)	0.0382 (3)	
H2	0.7781	0.2748	0.7373	0.046*	
C3	0.68030 (11)	0.16836 (17)	0.59459 (13)	0.0439 (3)	
H3A	0.6350	0.2794	0.6032	0.053*	
H3B	0.6930	0.1473	0.5056	0.053*	
C4	0.62021 (9)	0.00055 (17)	0.65005 (11)	0.0374 (3)	
H4	0.5530	−0.0204	0.6010	0.045*	
C5	0.68715 (10)	−0.18238 (16)	0.65113 (11)	0.0363 (3)	
H5	0.6573	−0.2689	0.5881	0.044*	
C6	0.80993 (10)	−0.15105 (16)	0.62629 (12)	0.0401 (3)	
H6A	0.8509	−0.2608	0.6528	0.048*	
H6B	0.8216	−0.1342	0.5367	0.048*	
C7	0.96943 (10)	0.0603 (2)	0.65718 (13)	0.0479 (3)	
C8	0.86994 (13)	0.3023 (2)	0.57707 (14)	0.0508 (4)	
C9	0.58864 (9)	0.0285 (2)	0.78582 (13)	0.0431 (3)	
C10	0.66715 (10)	−0.2597 (2)	0.78074 (13)	0.0434 (3)	

### Atomic displacement parameters (Å^2^)

**Table:**

	*U*^11^	*U*^22^	*U*^33^	*U*^12^	*U*^13^	*U*^23^
O1	0.0325 (5)	0.0985 (10)	0.0825 (9)	0.0007 (5)	−0.0011 (5)	0.0044 (7)
O2	0.0524 (6)	0.0621 (7)	0.0605 (7)	−0.0161 (5)	0.0189 (5)	−0.0008 (5)
O3	0.1034 (10)	0.0551 (7)	0.0736 (8)	−0.0151 (7)	0.0130 (7)	0.0214 (6)
O4	0.0493 (6)	0.0756 (8)	0.0679 (7)	0.0162 (5)	0.0054 (5)	−0.0189 (6)
O5	0.0465 (5)	0.0679 (7)	0.0369 (5)	0.0015 (4)	0.0045 (4)	0.0062 (4)
O6	0.0716 (7)	0.0576 (7)	0.0694 (7)	0.0034 (5)	0.0005 (6)	0.0278 (6)
C1	0.0332 (5)	0.0456 (6)	0.0326 (5)	−0.0024 (4)	0.0015 (4)	0.0029 (5)
C2	0.0437 (6)	0.0349 (6)	0.0359 (6)	−0.0044 (4)	0.0042 (5)	−0.0022 (4)
C3	0.0494 (7)	0.0388 (6)	0.0436 (7)	0.0024 (5)	−0.0066 (5)	0.0077 (5)
C4	0.0318 (5)	0.0427 (6)	0.0377 (6)	0.0014 (4)	−0.0066 (4)	0.0019 (5)
C5	0.0359 (6)	0.0361 (6)	0.0368 (6)	−0.0030 (4)	0.0003 (4)	0.0013 (4)
C6	0.0365 (6)	0.0379 (6)	0.0461 (7)	0.0009 (4)	0.0073 (5)	−0.0005 (5)
C7	0.0383 (6)	0.0595 (8)	0.0460 (7)	−0.0094 (6)	0.0031 (5)	−0.0051 (6)
C8	0.0627 (9)	0.0427 (7)	0.0471 (7)	−0.0126 (6)	0.0090 (6)	0.0006 (6)
C9	0.0282 (5)	0.0548 (7)	0.0464 (7)	0.0024 (5)	−0.0005 (5)	−0.0042 (5)
C10	0.0368 (6)	0.0487 (7)	0.0447 (7)	−0.0040 (5)	−0.0011 (5)	0.0091 (5)

### Geometric parameters (Å, º)

**Table:**

O1—C7	1.1850 (19)	C2—C3	1.5322 (18)
O2—C7	1.3745 (19)	C2—H2	0.9800
O2—C8	1.391 (2)	C3—C4	1.5226 (17)
O3—C8	1.1869 (19)	C3—H3A	0.9700
O4—C9	1.1923 (17)	C3—H3B	0.9700
O5—C9	1.3754 (18)	C4—C9	1.5070 (18)
O5—C10	1.3853 (18)	C4—C5	1.5390 (16)
O6—C10	1.1898 (18)	C4—H4	0.9800
C1—C7	1.5095 (17)	C5—C10	1.5039 (17)
C1—C6	1.5304 (17)	C5—C6	1.5341 (17)
C1—C2	1.5328 (17)	C5—H5	0.9800
C1—H1	0.9800	C6—H6A	0.9700
C2—C8	1.5068 (18)	C6—H6B	0.9700
			
C7—O2—C8	110.60 (10)	C5—C4—H4	108.4
C9—O5—C10	110.53 (10)	C10—C5—C6	111.75 (10)
C7—C1—C6	109.29 (10)	C10—C5—C4	103.39 (10)
C7—C1—C2	103.86 (10)	C6—C5—C4	112.99 (9)
C6—C1—C2	112.36 (10)	C10—C5—H5	109.5
C7—C1—H1	110.4	C6—C5—H5	109.5
C6—C1—H1	110.4	C4—C5—H5	109.5
C2—C1—H1	110.4	C1—C6—C5	111.25 (10)
C8—C2—C3	111.02 (11)	C1—C6—H6A	109.4
C8—C2—C1	103.55 (10)	C5—C6—H6A	109.4
C3—C2—C1	114.98 (10)	C1—C6—H6B	109.4
C8—C2—H2	109.0	C5—C6—H6B	109.4
C3—C2—H2	109.0	H6A—C6—H6B	108.0
C1—C2—H2	109.0	O1—C7—O2	120.18 (13)
C4—C3—C2	110.96 (9)	O1—C7—C1	129.62 (14)
C4—C3—H3A	109.4	O2—C7—C1	110.16 (12)
C2—C3—H3A	109.4	O3—C8—O2	121.05 (14)
C4—C3—H3B	109.4	O3—C8—C2	129.08 (16)
C2—C3—H3B	109.4	O2—C8—C2	109.86 (12)
H3A—C3—H3B	108.0	O4—C9—O5	120.41 (13)
C9—C4—C3	112.95 (11)	O4—C9—C4	129.29 (13)
C9—C4—C5	103.95 (10)	O5—C9—C4	110.30 (10)
C3—C4—C5	114.57 (10)	O6—C10—O5	119.85 (13)
C9—C4—H4	108.4	O6—C10—C5	129.67 (14)
C3—C4—H4	108.4	O5—C10—C5	110.48 (11)
			
C7—C1—C2—C8	13.48 (13)	C6—C1—C7—O2	109.53 (12)
C6—C1—C2—C8	−104.52 (11)	C2—C1—C7—O2	−10.57 (13)
C7—C1—C2—C3	134.77 (11)	C7—O2—C8—O3	−172.68 (15)
C6—C1—C2—C3	16.77 (14)	C7—O2—C8—C2	6.61 (15)
C8—C2—C3—C4	155.23 (11)	C3—C2—C8—O3	42.5 (2)
C1—C2—C3—C4	38.11 (15)	C1—C2—C8—O3	166.44 (16)
C2—C3—C4—C9	63.98 (13)	C3—C2—C8—O2	−136.70 (12)
C2—C3—C4—C5	−54.81 (14)	C1—C2—C8—O2	−12.78 (14)
C9—C4—C5—C10	11.25 (11)	C10—O5—C9—O4	−176.99 (12)
C3—C4—C5—C10	134.99 (11)	C10—O5—C9—C4	3.01 (13)
C9—C4—C5—C6	−109.73 (11)	C3—C4—C9—O4	45.97 (18)
C3—C4—C5—C6	14.02 (14)	C5—C4—C9—O4	170.76 (14)
C7—C1—C6—C5	−172.96 (10)	C3—C4—C9—O5	−134.04 (11)
C2—C1—C6—C5	−58.22 (13)	C5—C4—C9—O5	−9.25 (12)
C10—C5—C6—C1	−74.30 (13)	C9—O5—C10—O6	−175.24 (13)
C4—C5—C6—C1	41.81 (13)	C9—O5—C10—C5	4.90 (14)
C8—O2—C7—O1	−179.25 (14)	C6—C5—C10—O6	−68.34 (18)
C8—O2—C7—C1	2.77 (15)	C4—C5—C10—O6	169.85 (14)
C6—C1—C7—O1	−68.20 (19)	C6—C5—C10—O5	111.50 (11)
C2—C1—C7—O1	171.70 (15)	C4—C5—C10—O5	−10.31 (13)

### Hydrogen-bond geometry (Å, º)

**Table:**

*D*—H···*A*	*D*—H	H···*A*	*D*···*A*	*D*—H···*A*
C1—H1···O3^i^	0.98	2.40	3.3384 (19)	159
C3—H3*B*···O6^ii^	0.97	2.58	3.429 (2)	146

Symmetry codes: (i) *x*, −*y*+1/2, *z*+1/2; (ii) *x*, −*y*−1/2, *z*−1/2.

## References

[bb1] Ando, S., Ueda, M., Kakimoto, M., Kochi, M., Takeichi, T., Hasegawa, M. & Yokota, R. (2010). *The Latest Polyimides: Fundamentals and Applications*, 2nd ed. Tokyo: NTS.

[bb2] Bruker (2007). *APEX2* and *SAINT-Plus* Bruker AXS Inc., Madison, Wisconsin, USA.

[bb3] Burnett, M. N. & Johnson, C. K. (1996). *ORTEPIII* Report ORNL-6895. Oak Ridge National Laboratory, Tennessee, USA.

[bb4] Hasegawa, M., Fujii, M., Uchida, A., Hirano, D., Yamaguchi, S., Takezawa, E. & Ishikawa, A. (2008). *Polym. Prep. Jpn*, **57**, 4031–4032.

[bb5] Hasegawa, M. & Horie, K. (2001). *Prog. Polym. Sci.* **26**, 259–335.

[bb6] Hasegawa, M., Horiuchi, M. & Wada, Y. (2007). *High Perform. Polym.* **19**, 175–193.

[bb7] Sheldrick, G. M. (1996). *SADABS* University of Göttingen, Germany.

[bb8] Sheldrick, G. M. (2008). *Acta Cryst.* A**64**, 112–122. 10.1107/S0108767307043930 18156677

[bb9] Spek, A. L. (2009). *Acta Cryst.* D**65**, 148–155. 10.1107/S090744490804362X PMC2631630 19171970

[bb10] Uchida, A., Hasegawa, M. & Manami, H. (2003). *Acta Cryst.* C**59**, o435–o438. 10.1107/s0108270103013362 12909771

